# A method of undifferenced ambiguity resolution for GPS+GLONASS precise point positioning

**DOI:** 10.1038/srep26334

**Published:** 2016-05-25

**Authors:** Wenting Yi, Weiwei Song, Yidong Lou, Chuang Shi, Yibin Yao

**Affiliations:** 1 Collaborative Innovation Center of Geospatial Technology, Wuhan University, Wuhan 430079, China; 2 GNSS Research Centre, Wuhan University, Wuhan 430079, China; 3 School of Geodesy and Geomatics, Wuhan University, Wuhan 430079, China

## Abstract

Integer ambiguity resolution is critical for achieving positions of high precision and for shortening the convergence time of precise point positioning (PPP). However, GLONASS adopts the signal processing technology of frequency division multiple access and results in inter-frequency code biases (IFCBs), which are currently difficult to correct. This bias makes the methods proposed for GPS ambiguity fixing unsuitable for GLONASS. To realize undifferenced GLONASS ambiguity fixing, we propose an undifferenced ambiguity resolution method for GPS+GLONASS PPP, which considers the IFCBs estimation. The experimental result demonstrates that the success rate of GLONASS ambiguity fixing can reach 75% through the proposed method. Compared with the ambiguity float solutions, the positioning accuracies of ambiguity-fixed solutions of GLONASS-only PPP are increased by 12.2%, 20.9%, and 10.3%, and that of the GPS+GLONASS PPP by 13.0%, 35.2%, and 14.1% in the North, East and Up directions, respectively.

Precise point positioning (PPP) technique does not require dense arranged reference stations to achieve static positions with accuracy of the mm–cm level and that of dynamic positioning to the cm–dm level. Thus, PPP has been widely used in geodetic survey, GNSS seismology, precise orbit determination of low-earth orbit satellites, and aerial photogrammetry^1^,^2^,^3^,^4^. In traditional PPP based on an undifferenced model, ambiguity is usually regarded as a real value. Gabor and Nerem^5^ first proposed the algorithm and the concept of fixing ambiguity of a single station. However, PPP ambiguity fixing is not realized because of the policy of selective availability. Given that the accuracy of orbit and clock corrections is promoted continuously, the technique for PPP ambiguity fixing has improved rapidly in recent years^6^,^7^,^8^,^9^,^10^,^11^,^12^. Ge *et al*.^6^ indicated that the uncalibrated phase delays for the single difference between satellites should contain similar fractional cycle biases (FCBs). Reference station network is used to estimate the wide-lane and narrow-lane FCBs based on the single difference between satellites. The estimated FCBs are broadcasted to PPP users, who first need to eliminate the FCB of the receiver using the single difference between satellites. The received FCBs based on the single difference between satellites are then used to eliminate the FCBs of the satellites. In this way, the solution based on PPP ambiguity fixing is obtained. Different from Ge’s method, Laurichesse^7^ *et al*. and Collins^8^ *et al*. proposed the integer clock model and decoupled clock model with undifferenced ambiguity-fixed solution respectively. In the two methods, the clock biases of the satellite and receiver are utilized to absorb the FCB of the satellite and receiver respectively. In this way, the integer nature of ambiguity can be restored and the PPP ambiguity fixing is realized. This method is known as integer-recovery clock (IRC). Geng^13^ analyzed and compared the methods for fixing ambiguity, namely, the method based on FCB and IRC and proven their equivalence.

When attempting PPP ambiguity fixing, Melbourne–Wübbena (MW) combination observations^14^,^15^,^16^ are adopted to fix the wide-lane ambiguity. However, given that the GLONASS system adopts the signal pattern of frequency division multiple access, the inter-frequency code bias (IFCBs) and the inter-frequency carrier phase bias (IFPB) will affect the MW combination observations and will further influence the wide-lane ambiguity fixing. Scholars have comprehensively studied the characteristic of inter-frequency bias and its influence on GLONASS ambiguity fixing^17^,^18^,^19^,^20^,^21^,^22^. Their results show that the IFPB is mainly related to the receiver type and has a linear relation with frequency. Residual error can be reduced to a few millimeters after calibration and has hardly influence on wide-lane ambiguity fixing. However, no obvious linear relationship exists between the IFCBs and the frequency; hence, no unified and effective correction model is known^18^,^23^,^24^,^25^,^26^. Reussner and Wanninger indicated that IFCBs are difficult to correct. They proposed a method based on wide-lane carrier phase observations and an ionosphere model to fix wide-lane ambiguity. However, their method depends on a high-accuracy ionosphere model. To ensure reliable wide-lane ambiguity fixing, the ionosphere model must have a prediction accuracy higher than 1.4 total electron content units^27^. Hence, Geng and Bock recommend that for Europe if only IGS GIMs are available, GLONASS PPP-AR is best carried out within an area spanning less than 800 km in both longitudinal and latitudinal directions^28^. Banville *et al*. selected two GLONASS reference satellites with adjacent frequency numbers for integer ambiguities definition and thus absorb the linear dependency of the narrow-lane IFCBs with respect to frequency^22^. However, this method disregards the nonlinear parts of the narrow-lane IFCBs, even though these nonlinear parts are non-negligible. Therefore, the IFCBs are critical for processing undifferenced GLONASS ambiguity fixing.

In this study, we assume that IFPB can be precisely corrected in advance based on the exiting studies. To address the influence of the GLONASS IFCBs on wide-lane ambiguity fixing, we propose an undifferenced ambiguity resolution method for GPS+GLONASS PPP. The data of reference network are used to estimate the IFCBs calibrations and the FCB products, which are then delivered to the client to implement GLONASS ambiguity fixing. The rest of this article is organized as follows: first introduces the ambiguity fixing method for PPP, then elaborates the influence of the IFCBs on GLONASS wide-lane ambiguity fixing and introduces how the IFCBs are processed in GLONASS ambiguity fixing, then the data of EUREF Permanent network (EPN) are used to verify examples, the corresponding conclusion is presented at the last section.

## PPP data processing for ambiguity resolution

In dual-frequency PPP, ionosphere-free combination of observations is adopted to eliminate the effect of the first-order ionospheric delay^1^,^2^. The pseudorange and carrier phase observation equation of ionosphere-free combination are written as follows:


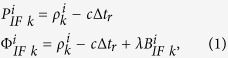


where *i* indicates the satellite number; *k* indicates the satellite number; 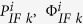 indicate the pseudorange and carrier phase observation of the ionosphere-free combination (meter); 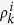 indicates the satellite–receiver geometric distance affected by errors, such as relativistic effects and earth tide; *c* indicates the speed of light; 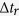 indicates the receiver clock error; *λ* indicates the wavelength of carrier phase ionosphere-free combination of observations; 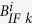 indicates the ambiguity of the ionosphere-free combination of observations (cycle), and it can be written as follows:


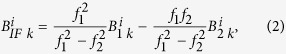


where 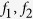 indicate the frequencies of carrier phase observation L1 and L2 respectively; and 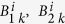 indicate the ambiguities of carrier phase observation L1 and L2 respectively. 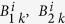 cannot be solved simultaneously when PPP ambiguity fixing is attempted. The ionosphere-free combination ambiguity is then generally expressed in the form of wide-lane ambiguity and narrow-lane ambiguity.


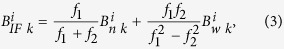


where 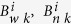 represents the wide-lane ambiguity and narrow-lane ambiguity, respectively. When attempting PPP ambiguity fixing, wide-lane ambiguity is initially fixed by MW combination observations, and then narrow-lane ambiguity is established by the fixed wide-lane integer ambiguity and the ionosphere-free combination ambiguity. Through narrow-lane ambiguity fixing, the fixed solution of ambiguity can be obtained for PPP^5^.


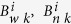
 in equation (3) are actually not integers because these are affected by the FCBs of the satellite and receiver^16^. Thus, the real-valued undifferenced wide-lane and narrow-lane ambiguities can be expressed as follows:


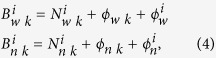


where 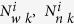 represent the wide-lane and narrow-lane integer ambiguities respectively; 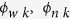 represent the wide-lane and narrow-lane FCBs in receivers; and 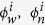 represent the wide-lane and narrow-lane FCBs in satellite respectively. The premise of the FCB-based ambiguity fixing method for PPP is using the observation data from the server’s reference network to separate FCB products that cause undifferenced ambiguity to become a non-integer. FCB products are then delivered to the client to restore the integer nature of the client’s undifferenced ambiguity as well as realize the search and fixation of undifferenced integer ambiguities at the client^6^.

### Effects of GLONASS narrow-lane IFCBs on wide-lane FCB estimation

Given that MW combination observations are irrelevant to the satellite–receiver geometric distance, with the first-order ionospheric delay eliminated^14^,^15^,^16^, MW combination observations are generally adopted to estimate wide-lane FCBs. The real-valued wide-lane ambiguity can be expressed as follows:


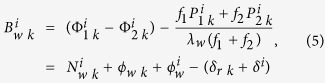


where 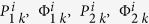 indicate the pseudorange and carrier phase observations at L1 and L2 respectively; and 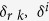 indicate the receiver and satellite pseudorange hardware delays respectively. The pseudorange and carrier phase multipath delays and the observation noise can be reduced through the averaging among multiple epochs and are negligible.

As shown in equation (5), wide-lane FCBs that are estimated by MW combination observations including the effects of the pseudorange-related hardware delays. For GPS, 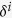 and 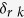 are relatively stable. Moreover, 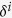 is the same for server and client stations, whereas 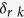 is the same for all satellites tracked at the receiver. Therefore, the characteristics of 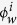 and 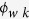
will not change if they are combined with 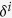 and 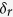 respectively. Accordingly, the following equation can be derived:


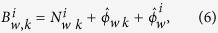


where 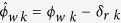, 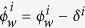. For the client, when wide-lane ambiguity is fixed through MW combination observations, the integer nature of wide-lane ambiguity can still be restored by using the FCB products^6^. However, for GLONASS, the pseudorange observation contains IFCBs that are currently difficult to correct^27^. Accordingly, the real-valued wide-lane ambiguity can be derived as follows:


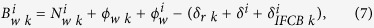


where 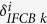 indicates the narrow-lane IFCBs^29^ and it should be noted that the IFPB is assumed to have been corrected in equation (7). In GLONASS, the estimation of wide-lane FCB by using MW combination observations will be affected by the narrow-lane IFCBs, as shown below.


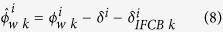


however, the IFCBs are related to several factors, such as receiver type and receiver firmware version. Hence, when the server and the client use different types of receivers, variations are observed between the narrow-lane IFCBs of the server and that of the client. If the narrow-lane IFCBs are ignored, then wide-lane FCB products estimated by the server will be unable to restore the integer nature of the undifferenced wide-lane ambiguity at the client-end. Wide-lane ambiguity fixing will then be affected. Therefore, narrow-lane IFCBs processing is the key to GLONASS ambiguity fixing. In the next section, the processing method of narrow-lane IFCBs in the wide-lane FCB estimation will be discussed.

### Narrow-lane IFCBs processing in wide-lane FCB estimation for GLONASS

In the FCB estimation, wide-lane FCB is generally estimated first. After wide-lane ambiguity is fixed, the narrow-lane ambiguity, which is composed of wide-lane ambiguity and ionosphere-free combination ambiguity, is used to estimate narrow-lane FCB. Ge *et al*.^6^ proposed that the substitution of wide-lane integer ambiguity can avoid the effect of wide-lane FCB estimation error on narrow-lane FCB estimation when the narrow-lane ambiguity is composed. According to equation (3), the following equation can be obtained:


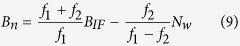


however, for GLONASS, the magnitude of the IFCBs can reach several meters at some frequencies^26^,^27^. This bias will not only affect wide-lane FCB estimation, but will also lead to integer deviation in wide-lane ambiguity fixing. However, the frequency ratio of GLONASS L1 and L2 observations is *f*_1_:*f*_2_ = 9:7. This ratio is substituted into equation (9), which derives the following equation:


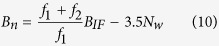


the wide-lane ambiguity’s coefficient is 3.5. For GLONASS, if the fixing of wide-lane ambiguity generates an integer-cycle deviation, and when integer-cycle deviations are odd, the narrow-lane ambiguity will have a half-cycle deviation, which will affect the narrow-lane FCB estimation. However, when integer-cycle deviations are even, the narrow-lane ambiguity will have integer deviation. This will have no influence on the narrow-lane FCB estimation. So, when fixing GLONASS ambiguity, the effects of fractional-cycle deviation (FRA-IFCB) and odd-cycle deviation (ODD-IFCB) on MW combination observations caused by the narrow-lane IFCBs need to be calibrated, whereas the absolute value of the narrow-lane IFCBs need not be corrected.

### Processing method of FRA-IFCB on MW combination observations

Considering the effects of the GLONASS IFCBs, the real-valued wide-lane ambiguity of the GLONASS satellite 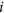 at station 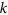 can be expressed as follows:


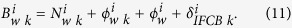


Fixing the real-valued wide-lane ambiguity to the nearest integer ambiguity derives the following equation:


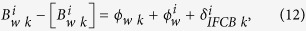


where 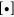 means taking the nearest integer. Assuming that *s* stations are present in the reference network, and *j* GLONASS satellites can be observed in each station, the following can be obtained:


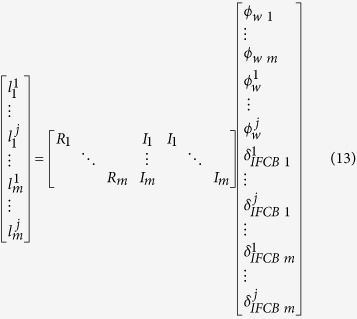


where 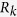 is an 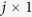 -order matrix with all elements being 1, 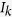 is an 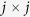 -order identity matrix, and 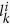 indicates the decimal part of the wide-lane ambiguity of the 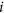 satellite at *k* station. In equation (12), the receiver FCB, satellite FCB, and IFCBs are linearly dependent. In equation (13), the normal equation is rank-deficient and needs the addition of 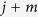 datum constraint equations. The present study selects one station as the base station because only single-differenced satellite FCB and the IFCBs between satellites make sense. This base station conforms to the following assumptions:

(1) The receiver FCB of base station is 0.

(2) The IFCBs of each satellite that can be observed at the base station is 0.

(3) The summation of the IFCBs of all satellites at the non-base station is 0.

The datum constraint equation can be expressed as follows:


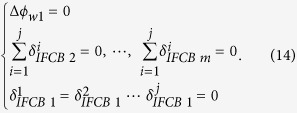


by adjusting the combined equations (13) and (14), the wide-lane FCBs of receiver and satellite and the IFCBs can be obtained. Notably, given that equation (12) eliminates the integer ambiguity parameter by rounding, only the FRA-IFCB can be obtained and the ODD-IFCB needs to be further corrected.

### Processing method of ODD-IFCB on MW combination observations

Supposing that after correcting the IFCBs for the station 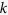, the wide-lane ambiguity no longer contains ODD-IFCB. From equation (10), the real-valued narrow-lane ambiguity of satellite 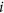 can be derived as


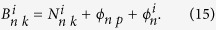


After correcting the FRA-IFCBs for the station *p*, if the wide-lane ambiguity still contains ODD-IFCB, then the real-valued narrow-lane ambiguity is as follows:


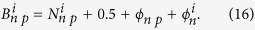


The station 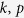 is made up of a baseline to evaluate the differences between satellites. The following can then be derived:


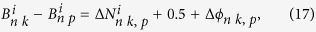


where 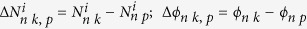. When the trigonometric function operation is used to eliminate the integer ambiguity parameter, the following can be obtained:


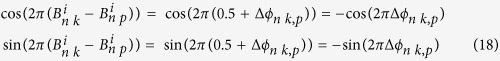



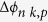
 represents the inter-station single-difference receiver FCB, which has the same effect on different satellites at the same baseline. This means that if the wide-lane ambiguity is fixed correctly, the sine and cosine values of real-valued narrow-lane ambiguities of different satellites, in theory, are equivalent. But as shown in equation (18), when the wide-lane ambiguity fixed for a satellite has even-cycle deviation, compared with the sine and cosine of other satellites, the values are equal but the signs are opposite. On this basis, the ODD-IFCB caused by narrow-lane IFCBs on the wide-lane ambiguity can be calibrated by the following steps:

(1) Selecting one station as the reference station and assume that the wide-lane ambiguity no longer contains ODD-IFCB at the reference station.

(2) Selecting another station which is near the reference station to make up a baseline. The trigonometric function operation is used to the inter-station single-difference real-value narrow-lane ambiguities. If equation (18) is obtained, it means the wide-lane ambiguity contains ODD-IFCB at the selecting station and the ODD-IFCB should be set to 1.

(3) Selecting other stations to make up baselines with the reference station or the stations which have been calibrated. Repeat the step 2) until all the stations having been calibrated.

To get more precise IFCB calibrations, the coordinates of the stations can be fixed and the post-process method can be used. As the IFCB is mainly related to receiver type and receiver firmware version^26^, the IFCB for different receiver type and firmware version can be estimated as well as the FCB estimation and then provided to clients. Anyway, the receiver type or firmware version of the client is probably not included in the reference network. In this situation, we can use the follow steps to obtain the IFCB calibrations.

(1) Assume that the receiver wide-lane FCB is zero, after correcting the satellite wide-lane FCBs by the wide-lane FCB products provided by the server, as shown in equation (12), the FRA-IFCB can be easily obtained.

(2) In equation (16), if the satellite narrow-lane FCB has been corrected, the remains are only integer ambiguity, receiver FCB, and the half-cycle deviation. Then the trigonometric function operation is used to eliminate the integer ambiguity. Similarly to equation (18), the ODD-IFCB should be set to 1, if the values of the sine and cosine are equal but signs are opposite.

Notably, the ODD-IFCB at the reference station will be absorbed into the narrow-lane FCB. But this will not affect the ambiguities fixing at client-end, as the datum of the wide- and narrow-lane FCB and IFCB products are unified at the server and client-end.

### Experimental validation

**Figure 1:**
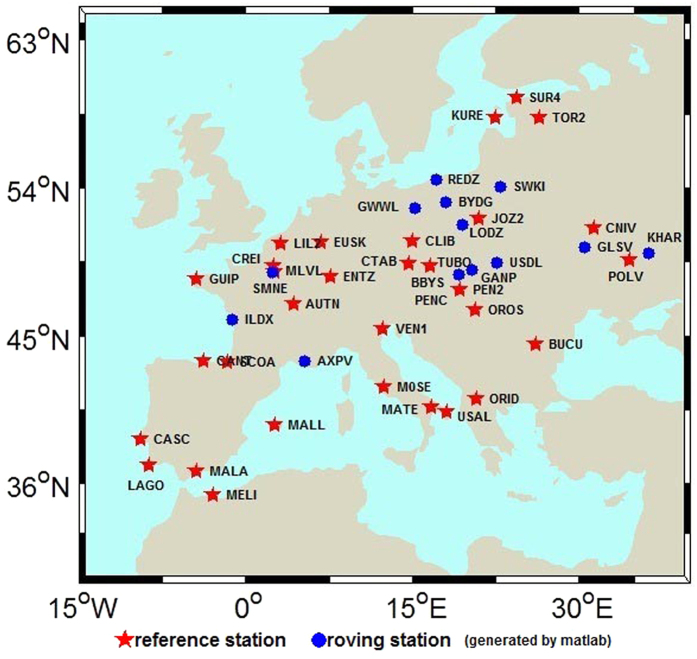
Station distribution. The average distance of the rover stations to their nearest reference stations is about 210 km, while the least distance is about 12 km and the biggest distance is about 393 km. This figure is drawn using Matlab V7.0 with M_Map V1.4h (https://www.eoas.ubc.ca/~rich/map.html).

The GPS+GLONASS data continuously observed by 45 stations (32 servers, 13 clients) of EPN for day of year (DOY) 335–341 in 2013 were adopted. The station distribution is shown in Fig. 1 and the receiver and antenna types and the firmware version are listed in Table 1. In Fig. 1, the red pentagrams represent reference stations and the blue cycles represent the clients. In this study, the precise orbits and clock error products with 30-second sampling interval provided by the European Space Agency were adopted; the errors such as earth tide and phase windup were corrected with the precise model provided by the International GNSS Service (IGS); and the antenna phase center was corrected with the absolute antenna phase center model provided by IGS. The GLONASS IFPB are corrected using the method proposed by Wanninger^20^ while the GLONASS IFCB are processed by the method proposed in this study. After the estimation of wide-lane and narrow-lane FCBs is completed by the server, these FCB products and IFCBs corrections are delivered to the client. To improve the FCB estimation precision, the server fixes station coordinates and the satellite’s elevation mask angle is set to 15°. The client adopts the dynamic PPP model and the same error correction model as the server. The integer nature of undifferenced ambiguity is restored by the FCB provided by the server and the corrected IFCBs. Through least-square ambiguity decorrelation adjustment (LAMBDA)^30^, the wide-lane and narrow-lane ambiguities are searched and fixed, followed by the fixing of ionosphere-free combination ambiguity. Thus, the ambiguity-fixed solution can be obtained for PPP. This study adopted partial ambiguity fixing strategy to fix the ambiguity and adopts the ratio and probability 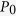 to check if the ambiguity can be fixed^31^,^32^. The thresholds of ratio and 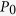 are 4 and 0.99 respectively. The calculation equation of probability 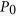 is as follows^32^:


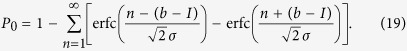


Where 
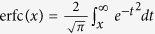
 and 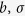 represent the float ambiguity and its accuracy respectively, and *I* is the nearest integer value of *b*.

**Figure 2:**
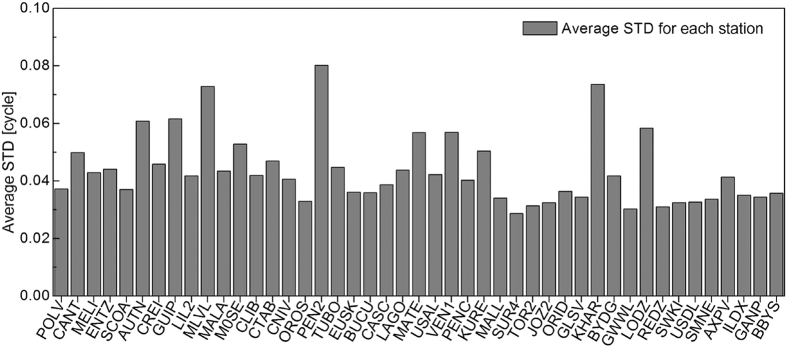
The average STD of the GLONASS IFCB estimations for each station in 30 days.

**Figure 3:**
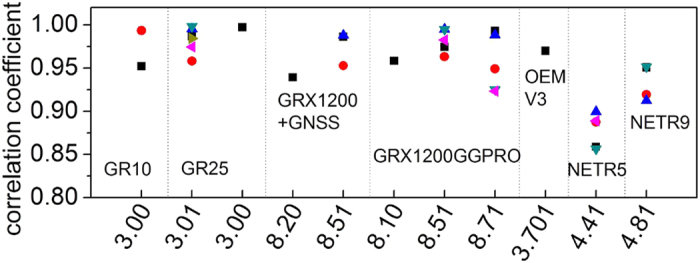
The correlation coefficient for each receiver type and firmware version. For each receiver type and firmware version, one station is selected as the reference, the correlation coefficients are the other stations to the reference station.

To analyze the stability of the IFCB estimation, 30 days data are used to estimate the IFCB (one parameter for each station and each satellite) and the standard deviations (STDs) of the IFCB for each station and each satellite are computed. For simplify data analysis, the average STDs of all 24 satellites for each station are obtained. Figure 2 shows the average STDs of each station. As shown in Fig. 2, the average STDs of all the station are superior to 0.08 cycle and that of most station are superior to 0.05 cycle. This means IFCB is stable and can be consider as the same over 30 days. So the average IFCB over 30 days is consider as the final results for each station. Then, the relationship between different stations is analyzed. Considering that IFCB is related to receiver type and firmware, so in this study, for each receiver type and firmware version, one station is selected as the reference, then the correlation coefficients of the other stations to the reference station are computed. Figure 3 show the correlation coefficient for each receiver type and firmware version. As shown in Fig. 3 the correlation coefficients of the Trimble NetR5 is about 0.85, while the others are all high up to 0.9, which means the stations with the same receiver type and firmware are strongly correlated even if the antenna type is different. Thus, when data are processed, IFCBs of the same receiver type and receiver firmware version can be considered as the same.

**Figure 4:**
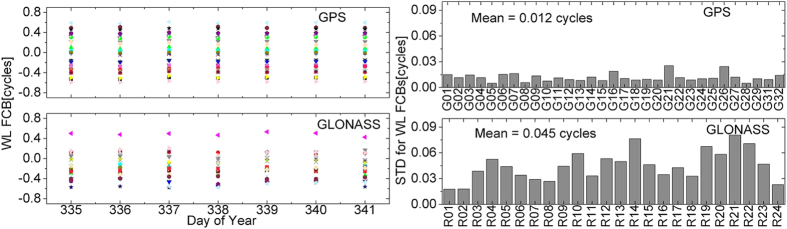
The repeatability of GPS and GLONASS wide-lane FCB estimations in one week. The left figure shows the FCB estimations of each satellite in seven days, and different colors represent different satellites. The right figure presents the STDs of FCB estimations in seven days, and the average STD is marked on the top left corner.

Figure 4 shows the wide-lane FCB estimations of GPS and GLONASS satellites in continuous observation duration of DOY 335–1 in 2013, as well as the standard deviation (STD) of FCB estimations of each satellite in seven days. The GPS and GLONASS wide-lane FCBs are relatively stable. The STDs of wide-lane FCB estimations of GPS satellites in seven days are all superior to 0.03 cycle, and the average STD is only 0.012 cycle. GLONASS stability is relatively lower. However, the STD of wide-lane FCB estimations of each satellite in seven days is superior to 0.09 cycle, and the average STD is superior to 0.05 cycle. It is illustrated that GPS and GLONASS wide-lane FCBs are relatively stable in a week and can be forecasted for several days.

**Figure 5:**
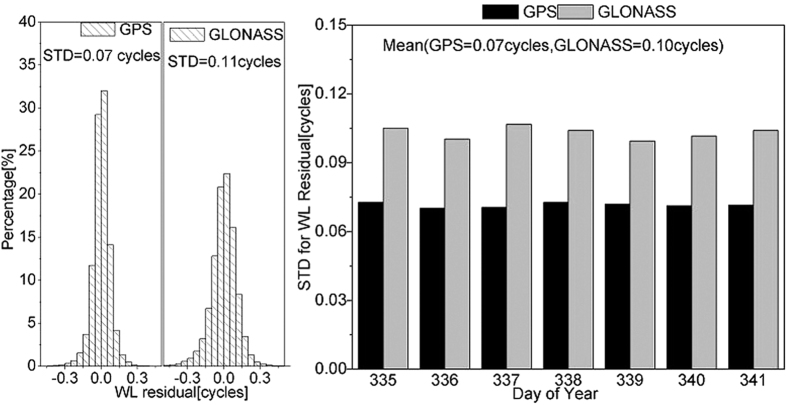
Residual posterior distribution of wide-lane FCB estimations on DOY 335 and STD values of wide-lane residuals in seven days. The left figure shows the residual posterior distribution with the provided STD values. The right figure shows the STD of wide-lane residuals in seven days.

The residual analysis after the ambiguity fixed by FCB products is an effective method to evaluate the FCB estimation accuracy^6^,^10^. This study used FCB estimation products to fix ambiguities of the reference network. After correcting the satellite and receiver FCBs, the ambiguity was fixed through rounding and the residual was analyzed. Figure 5 lists the residuals after GPS and GLONASS wide-lane ambiguities are fixed and the STD values of residuals in seven days. GPS and GLONASS wide-lane residuals present normal distribution. Around 99.1% of GPS wide-lane residuals are less than 0.25 cycle, and around 96.9% of GLONASS residuals are less than 0.25 cycle. After correcting wide-lane FCB, the wide-lane ambiguity is very close to the integer value. With 0.25 cycle as the threshold, most wide-lane ambiguities of GPS and GLONASS satellites can be fixed by wide-lane FCB products. Based on the STD of wide-lane residuals in seven days, the STDs of GPS and GLONASS wide-lane residuals in different days are also closer. The effects of wide-lane ambiguity fixing among different days are all favorable.

**Figure 6:**
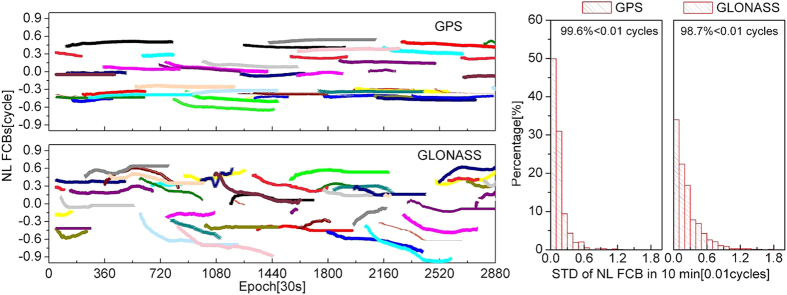
Time series diagram of GPS and GLONASS narrow-lane FCB estimations of DOY 335 in 2013 and STDs of narrow-lane FCB estimations in 10 min. The left figure shows the time series diagram, and different colors represent different satellites. The right figure shows STD statistics of narrow-lane FCB estimations in 10 min.

Figure 6 shows the time series of GPS and GLONASS narrow-lane FCB estimations and the statistics of the STDs of narrow-lane FCB estimations in every 10 minutes. As shown in this figure, the narrow-lane FCB estimations are affected by residuals such as clock error and atmospheric delay and change over time in one day, with the STD can reaching about 0.3 cycle. The change of GLONASS narrow-lane FCBs is larger than that of GPS narrow-lane FCBs. Although narrow-lane FCBs are changed considerably in seven days, the change is slow. The STD statistics in 10 min indicate that around 99.6% of the GPS STDs are less than 0.01 cycle, and the largest STD is only 0.012 cycle. Around 98.7% of the GLONASS STDs are less than 0.01 cycle, and the largest STD is around 0.017 cycle. In 10 min, narrow-lane FCBs are relatively stable and can be considered constant.

**Figure 7:**
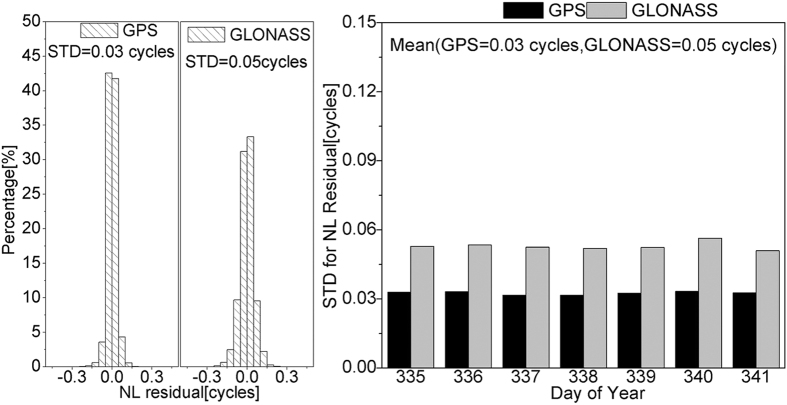
Residual posterior distribution of narrow-lane FCB estimations on DOY 335 and STD values of narrow-lane residuals in seven days. The left figure shows the residual posterior distribution and the STD values of residuals. The right figure shows STD values of narrow-lane residuals in seven days.

The residuals after fixing GPS and GLONASS narrow-lane ambiguities of DOY 335 in 2013 are calculated as well as the STD values of narrow-lane residuals in one week, as shown in Fig. 7. For GPS, 98.5% of the residuals of narrow-lane FCBs are less than 0.10 cycle, and 99.7% are less than 0.15 cycle; for GLONASS, around 93.5% of the residuals of narrow-lane FCBs are less than 0.10 cycle, and 98.7% are less than 0.15 cycle. When GPS and GLONASS narrow-lane ambiguities are fixed, the residuals are smaller. This confirm that the narrow-lane ambiguity fixing is reliable and the proposed method can effectively eliminate the effects of the GLONASS IFCBs to realize GLONASS ambiguity fixing. However, GLONASS residuals are still relatively large compared with GPS residuals. The first reason may be that the precision of GLONASS orbits and clock error products is slightly worse than the precision of GPS. And the second reason maybe that, for GLONASS, the IFPBs cannot be totally eliminated in this study and the IFPB residuals will affect the accuracy of the narrow-lane FCBs.

**Figure 8:**
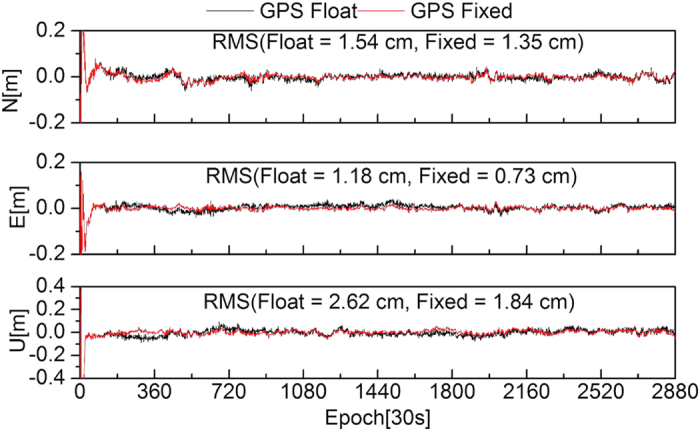
Positioning time series of PPP float solutions and fixed solutions of GPS-only at the SWKI station.

**Figure 9:**
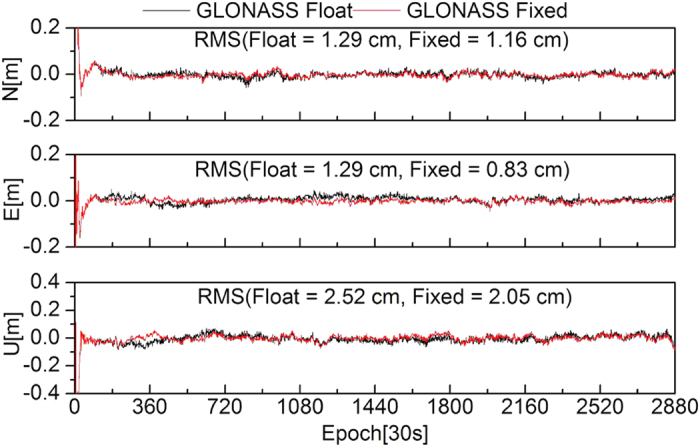


**Figure 10:**
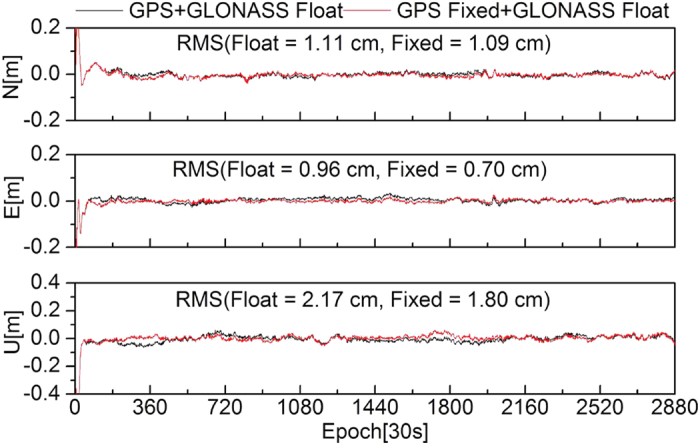
Positioning time series of PPP float solutions and fixed solutions (only fixing the GPS ambiguities) of the combined GPS+GLONASS at the SWKI station.

**Figure 11:**
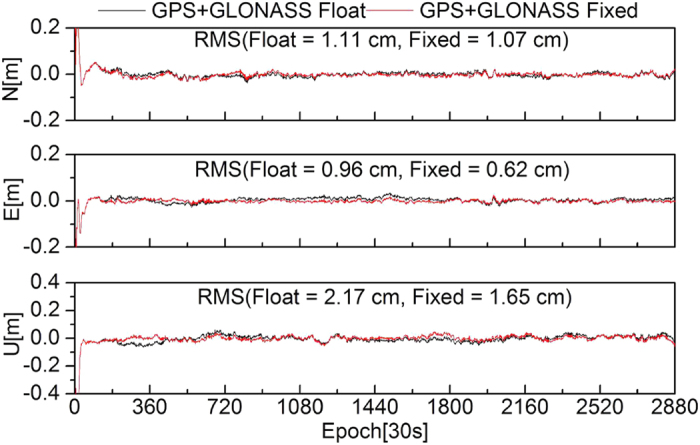
Positioning time series of PPP float solutions and fixed solutions of the combined GPS+GLONASS at the SWKI station.

To further evaluate the effectiveness of the ambiguity fixing method in this study, 13 stations were selected to analyze the performance of PPP ambiguity-fixed solutions of GPS-only, GLONASS-only, and combined GPS+GLONASS at the client. The true-value coordinates of the stations were provided by weekly SINEX solutions. Figures 8, 9, 10, 11 show the positioning time series of PPP ambiguity float and fixed solutions of GPS-only, GLONASS-only, and combined GPS+GLONASS at the SWKI station on DOY 335 in 2013. Ambiguities of the GPS and GLONASS can be fixed using FCB products estimated in this study. After fixing ambiguities, the dynamic PPP accuracies of GPS-only, GLONASS-only, and the combined GPS+GLONASS can all be improved.

**Figure 12:**
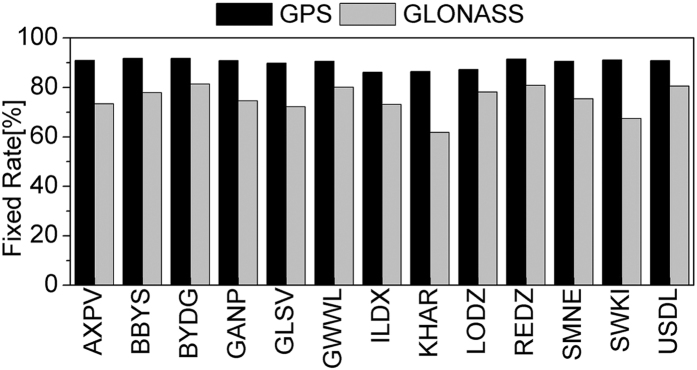
Average fixing rates of GPS and GLONASS ambiguities at each station.

**Figure 13:**
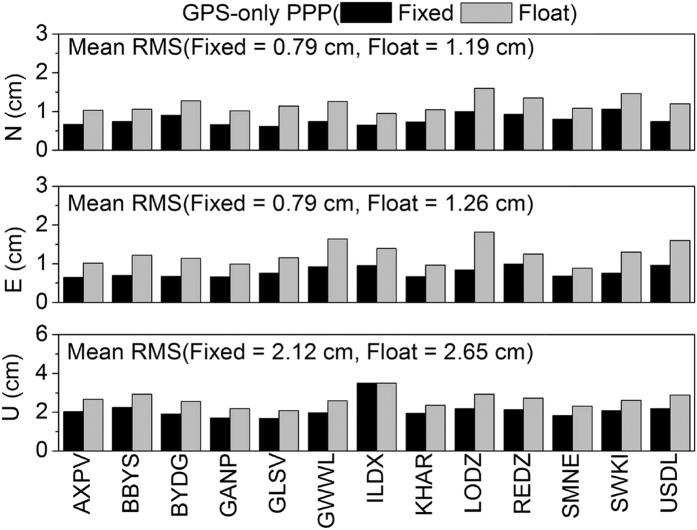
Average RMS of ambiguity float and fixed solutions of GPS-only PPP in seven days.

**Figure 14:**
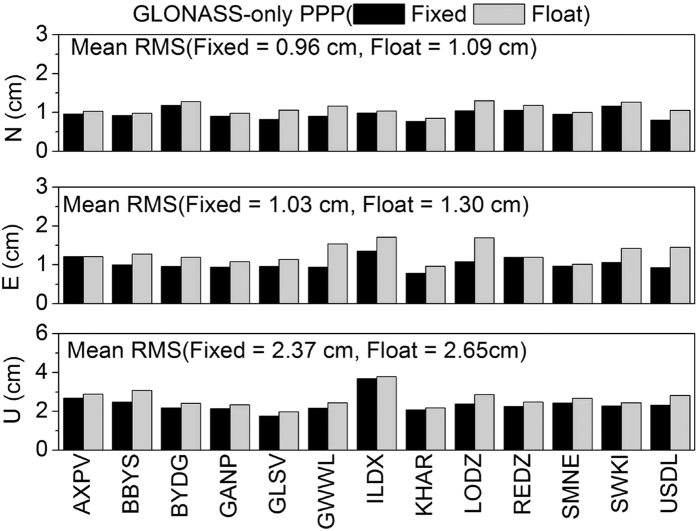
Average RMS of ambiguity float and fixed solutions of GLONASS-only PPP in seven days.

**Figure 15:**
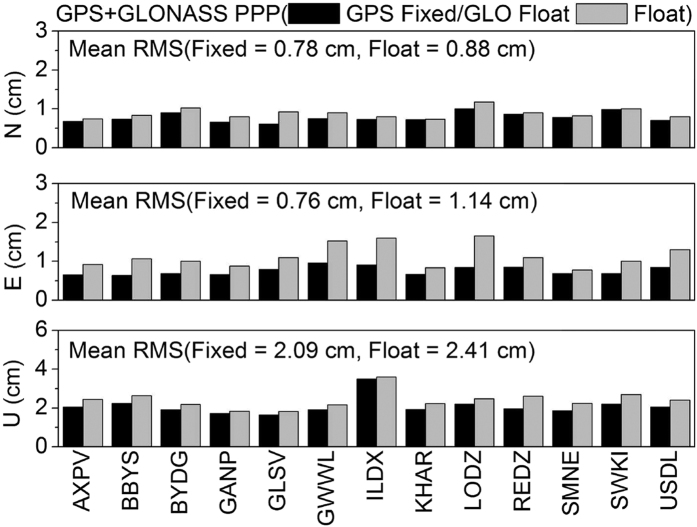
Average RMS of ambiguity float and fixed (only fixing the GPS ambiguities) solutions of the combined GPS+GLONASS PPP in seven days.

**Figure 16:**
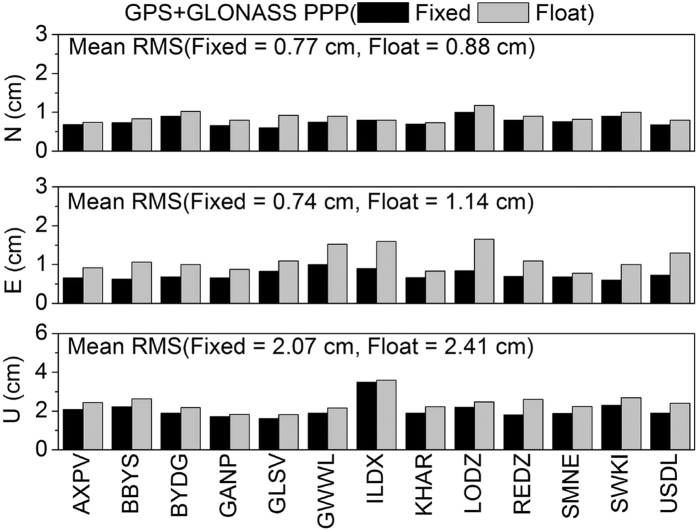
Average RMS of ambiguity float and fixed solutions of the combined GPS+GLONASS PPP in seven days.

Figure 12 shows the average fixing rates of GPS and GLONASS ambiguities at 13 stations in seven days. The fixing rate is around 90% for GPS, but only around 75% for GLONASS. Figures 13, 14, 15, 16 show the average root mean square (RMS) of ambiguity float and fixed solutions of GPS-only, GLONASS-only, and the combined GPS+GLONASS PPP at each station on DOY 335–341 in 2013. After ambiguities are fixed, the positioning accuracy of each station is improved. The average RMS GPS-only PPP at three directions (N, E, U) is reduced respectively from 1.19, 1.26, and 2.65 cm to 0.79, 0.79, and 2.12 cm; the accuracy in the three directions increased by 33.9%, 37.6%, and 20.2%. The average RMS of GLONASS-only at three directions (N, E, U) is reduced respectively from 1.09, 1.30, and 2.65 cm to 0.96, 1.03 and 2.37 cm; the accuracy in the three directions increased by 12.2%, 20.9%, and 10.3%. Compared with that for GPS, the ambiguity fixed for GLONASS contributes less to the accuracy improvement. This result is probably because GLONASS has a lower fixing rate than GPS, and the accuracy of GLONASS narrow-lane FCB estimation is relatively lower than that of GPS. For the combined GPS+GLONASS PPP, if only GPS ambiguities are fixed, the average RMS at three directions (N, E, U) is reduced respectively from 0.88, 1.14, and 2.41 cm to 0.78, 0.76, and 2.09 cm; the accuracy in the three directions increased by 11.7%, 33.3%, and 13.3%. And if both the GPS and GLONASS ambiguities are fixed, the average RMS is reduced to 0.77, 0.74, and 2.07 cm, increasing by 13.0%, 35.2%, and 14.1%. It can be seen that, for combined GPS+GLONASS PPP, fixing both the GPS and GLONASS ambiguities contributes less to the accuracy improvement than that fixing only GPS ambiguities. This is because if GPS ambiguities can be well fixed, generally, there will be more than seven ambiguities being fixed. The position accuracy can well be improved. In this situation, fixing GLONASS ambiguities will contributes less to the accuracy improvement.

## Conclusions

This study proposes undifferenced ambiguity resolution methods for GPS+GLONASS PPP, which consider the IFCBs estimation. This study also establishes the parameter estimation models for the IFCBs at the server. The client can utilize the satellite wide-lane and narrow-lane FCBs delivered by the server as well as IFCBs products to eliminate the effect of the GLONASS IFCBs. The example result demonstrates that IFCB are very stable over 30 days and the same receiver type and firmware are strongly correlated. Using the IFCB corrections, GLONASS undifferenced ambiguity can be fixed and the success rate can reach 75%. Compared with the ambiguity float solutions, the positioning accuracies of ambiguity-fixed solutions of GLONASS-only PPP at N, E, and U directions are increased respectively by 12.2%, 20.9%, and 10.3%; the accuracies of ambiguity-fixed solutions of the combined GPS+GLONASS PPP are increased by 13.0%, 35.2%, and 14.1% in the three directions. However, the method proposed in this article requires the IFCBs corrections of different types of receivers. The client need to select the IFCB corrections according their receiver type and firmware version and calculate their own IFCB corrections if their receiver type or firmware version is not included in table list of the IFCB corrections. Furthermore, apart from the selected receivers of Leica series, Nov type, and Trimble series in this article, more types of receivers need to be used for example verification^33^.

## Additional Information

**How to cite this article**: Yi, W. *et al*. A method of undifferenced ambiguity resolution for GPS+GLONASS precise point positioning. *Sci. Rep.* **6**, 26334; doi: 10.1038/srep26334 (2016).

## Figures and Tables

**Table 1:** The receiver and antenna types as well as the firmware version.

**Station**	**Manufacturer**	**Receiver Type**	**Antenna Type**	**Firmware version**
Server	Leica	GR10	LEIAR10	3.00
			LEIAR25.R4	
		GR25	TRM55971.00	3.01
			LEIAR25.R4	3.00
		GRX1200+GNSS	LEIAR25.R4	8.20
			LEIAR10	8.51
			LEIAR25.R3	
			LEIAR25.R4	
		GRX1200GGPRO	LEIAT504GG	8.10
			LEIAT504GG	8.51
Client	NOV	OEM3	NOV702GG	3.701
	Trimble	NetR5	TRM55971.00	4.41
		NetR9	TRM55971.00	4.81
			TRM59800.00	
